# Oviposition Preferences of the Fall Armyworm (*Spodoptera frugiperda*) (Lepidoptera: Noctuidae) in Response to Various Potential Repellent and Attractant Plants

**DOI:** 10.3390/insects15110885

**Published:** 2024-11-13

**Authors:** Kervin Can, Tsui-Ying Chang, Lekhnath Kafle, Wen-Hua Chen

**Affiliations:** 1 Department of Tropical Agriculture and International Cooperation, National Pingtung University of Science and Technology, 1 Xuefu Road, Neipu, Pingtung 912, Taiwan; kervincan@gmail.com (K.C.); kafle@mail.npust.edu.tw (L.K.); 2 Department of Plant Medicine, National Pingtung University of Science and Technology, 1 Xuefu Road, Neipu, Pingtung 912, Taiwan; tychang@mail.npust.edu.tw

**Keywords:** desmodium, egg laying, host plant suitability, integrated pest management, intercropping, para grass, push-pull strategy, reduced oviposition effects, sunhemp

## Abstract

The fall armyworm (FAW), *Spodoptera frugiperda*, is a major polyphagous pest that mainly feeds on maize and other cash crops. Understanding *S. frugiperda*’s behavior on different host plants facilitates the development of effective integrated pest management (IPM) plans. Therefore, this study investigated the oviposition preferences of *S. frugiperda* females among different host plants using no-choice, two-choice, and multiple-choice bioassays. In no-choice bioassays, para grass, *Urochloa mutica* (Forssk.) (Poales: Poaceae); maize, *Zea mays* (L.) (Poales: Poaceae); and napier grass, *Pennisetum purpureum* (Schumach) (Poales: Poaceae) were identified as highly attractive, while sweet sorghum, *Sorghum dochna* (Forssk.) (Poales: Poaceae); sunhemp, *Crotalaria juncea* (L.) (Fabales:Fabacea); Egyptian clover, *Trifolium alexandrinum* (L.) (Fabales:Fabacea); desmodium, *Desmodium uncinatum* (Jacq.) (Fabales:Fabacea); natal grass, *melinis repens* (Zizka) (Poales: Poaceae); molasses grass, *Melinis minutiflora* (P.Beauv.) (Poales: Poaceae); and mung bean, *Vigna radiata* (R. wilczek) (Fabales: Fabaceae) exhibited reduced oviposition effects. Two-choice bioassays revealed different levels of attractiveness and repellency among different plant combinations. In multiple-choice bioassays, mimicking an intercropping scenario, differences in the number of eggs and egg mass were observed for M:S:D:W (maize, sunhemp, desmodium, and cage wall), S:D:M:W (sunhemp, desmodium, maize, and cage wall), and D:M:S:W (desmodium, maize, sunhemp, and cage wall). This study provides insights into the egg-laying preferences of *S. frugiperda* females among different host plants, valuable for the management of *S. frugiperda*. This encourages further research and further identification of novel repellent and attractant host plants, which will ultimately contribute to the development of sustainable and environmentally friendly crop production practices and techniques.

## Simple Summary

The fall armyworm (FAW), *Spodoptera frugiperda* (Lepidoptera: Noctuidae), has caused significant crop damage worldwide, particularly to maize and other economically important crops. While chemical control remains common, understanding pest behavior and host plant preferences could enable more sustainable management approaches. This study investigated the oviposition preferences of *S. frugiperda* among various host plants using no-choice, two-choice, and multiple-choice bioassays. In no-choice bioassays, para grass, maize, and napier grass were highly attractive for *S. frugiperda* oviposition, while plants like sweet sorghum, sunhemp, desmodium, Egyptian clover, molasses grass and mung bean were less preferred. Two-choice bioassays revealed differing levels of attractiveness and repellency among plant combinations. In multiple-choice experiments simulating intercropping scenarios, the number of *S. frugiperda* eggs and egg masses varied across maize, sunhemp, desmodium, and the cage walls. These findings suggest that strategic intercropping of attractive and less preferred host plants could be a promising approach to disrupt *S. frugiperda* oviposition and reduce subsequent larval infestations. This study provides valuable insights into the ovipositional behavior of *S. frugiperda* and highlights the potential of intercropping as an environmentally friendly pest management strategy. By reducing reliance on chemical pesticides, such approaches can contribute to sustainable agriculture and protect crop yields.

## 1. Introduction

In the search for a suitable host, lepidopterans rely on volatile chemical cues released by host plants to find suitable habitats, food, and oviposition [1,2,3,4]. These chemical cues are emitted by plants as a defense mechanism, and can range from simple organic compounds to complex mixtures of molecules [5]. Over time, these defense mechanisms can be severely affected by agricultural selection and domestication, compromising the ability of the plant to defend itself. A typical example of this scenario is the breeding process of maize, which has led to the loss of its protective characteristics, with modern varieties being more affected than local genotypes.

As plant breeding processes continue with the selection of high-yielding genotypes, one pest, in particular, has taken advantage of this vulnerability. The fall armyworm *Spodoptera frugiperda* (J. E. Smith) (Lepidoptera: Noctuidae) is an extremely voracious and polyphagous pest. It is native to the tropical regions of the Western Hemisphere, from the United States to Argentina, and causes considerable damage to maize and many other crops. This invasive species was first reported in North America, and later in many African countries in early 2016. Subsequently, South Asia reported its first sighting in 2018, particularly in India, followed by other countries such as Sri Lanka, Bangladesh, Myanmar, Thailand, and China [6].

Economically important pests are usually controlled using chemical methods. However, the use of chemical pesticides to control *S. frugiperda* faces several challenges, including inappropriate use, unaffordability, the development of resistance by the targeted pest, and potential harm to both humans and the environment [7,8,9]. This has, in part, led to a shift in interest towards integrated pest management (IPM) strategies that are cost-effective, environmentally friendly, and practical for small-scale farmers. A promising IPM strategy is push-pull, in which trap and repellent plants are used to repel or attract pests [10,11].

Previous studies have investigated various attractant and repellent host plants, such as napier grass, *Pennisetum purpureum* (Schumach) (Poales: Poaceae); sudan grass, *Sorghum sudanense* (Piper, Stapf) (Poales: Poaceae); and *Brachiaria* cv Mulato II (Poales: Poaceae), which have been shown to be effective in attracting more stem borer moths compared to maize [12,13]. Further studies have focused on various repellent crops to minimize *S. frugiperda* infestation and crop damage. Likewise, they studied the repellent properties of plants such as marigold, *Tagetes erecta* (L.) (Asterales: Asteraceae); molasses grass, *Melinis minutiflora* (P.beauv.) (Poales: Poaceae); and two species of plants belonging to the genus desmodium, namely silverleaf desmodium, *Desmodium uncinatum* (Jacq.) (Fabales:Fabaceae) and green leaf desmodium, *Desmodium intortum* (Mill.) (Fabales:Fabaceae). These plants have been found to exhibit repellency against *Busseola fusca* (Fuller) (Lepidoptera: Noctuidae) and *S. frugiperda* [3,12]. In addition, intercropping methods involving the cultivation of crops, such as maize with legumes or cereals, have also been examined, as these combinations can disrupt the host-seeking behavior of *S. frugiperda* and limit its population growth [14,15,16].

Although several studies have reported that the preferred repellent intercrop in Africa to manage *S. frugiperda* is desmodium [17], access to quality seeds (Taiwan, East Asia) has been problematic; slow growth is another issue; and third, it is a delicate plant that is difficult to manage. Therefore, to improve this management strategy further, alternative attractant and repellent host plants that are readily available, easy to manage, and rapidly growing must be evaluated and selected.

Hence, this study aimed to evaluate the oviposition preferences of potential attractants and easily accessible repellent host plants. The oviposition preferences of these plants were evaluated under laboratory conditions by using no-choice, two-choice, and multiple-choice bioassays to determine their attractant and repellent properties. Egg masses oviposited on potential attractant and repellent plants were collected and counted every 24 h. The results were analyzed to determine the increased or reduced oviposition effects of the plants.

Ultimately, the development of IPM strategies that are suitable for small farmers is essential for the effective control and management of *S. frugiperda*. Push-pull strategy using attractant and repellent host plants is a promising strategy for managing *S. frugiperda*. The results of this study will help identify potential host plants for the management of *S. frugiperda* in Taiwan and other parts of the world, and contribute to the development of cost-effective, environmentally friendly, practical, and sustainable IPM strategies.

## 2. Materials and Methods

### 2.1. Test Plants

In this experiment, 12 potential host plants were used: napier grass, *Pennisetum purpureum* (Schumach) (Poales: Poaceae); nill grass, *Acroceras macrum* (Stapf) (Poales: Poaceae); natal grass, *melinis repens* (Zizka) (Poales: Poaceae); sunhemp, *Crotalaria juncea* (L.) (Fabales: Fabaceae); sweet sorghum, *Sorghum dochna* (Forssk.) (Poales: Poaceae); greenleaf desmodium, *Desmodium uncinatum* (Jacq.) (Fabales:Fabacea); Egyptian clover, *Trifolium alexandrinum* (L.) (Fabales:Fabaceae); molasses grass, *Melinis minutiflora* (P.Beauv.) (Poales: Poaceae); maize, *Zea mays* (L.) (Poales: Poaceae); para grass, *Urochloa mutica* (Forssk.) (Poales: Poaceae); faba beans, *Vicia faba* (L.) (Fabales: Fabaceae); and mung bean *Vigna radiata* (R. wilczek) (Fabales: Fabaceae). Thirteen-centimeter pots were filled with soil and transferred to a greenhouse. Seeds and cuttings were planted directly into each pot. General maintenance included watering and fertilization. The plants were allowed to grow to the 4–6 leaf stage before being utilized for oviposition bioassay purposes.

### 2.2. Insect Colony

Egg masses and larvae were collected at the Sustainable Agriculture Research Farm located at the National Pingtung University of Science and Technology campus (22°38′50.1″ N, 120°37′08.1″ E). To ensure the optimal development and purity of the egg masses and larvae, they were maintained and reared under laboratory conditions at 27 °C, 70–75% relative humidity (RH), and 14:10 h (L:D) for more than five generations before being used for the oviposition preference bioassays. The neonates were fed an artificial diet modified from Midega et al. [18]. Field-collected larvae were individually reared in petri dishes (100 × 15 mm) and fed daily with fresh maize leaves. The field-collected larvae were fed fresh maize leaves, as previous studies have shown that some larvae die after consuming an artificial diet. At the adult stage, they were sexed, placed into 90 L × 44 W × 44 H cm insect-rearing net cages, and fed a 10% honey solution.

### 2.3. Oviposition Preference Bioassays

**Figure 1:**
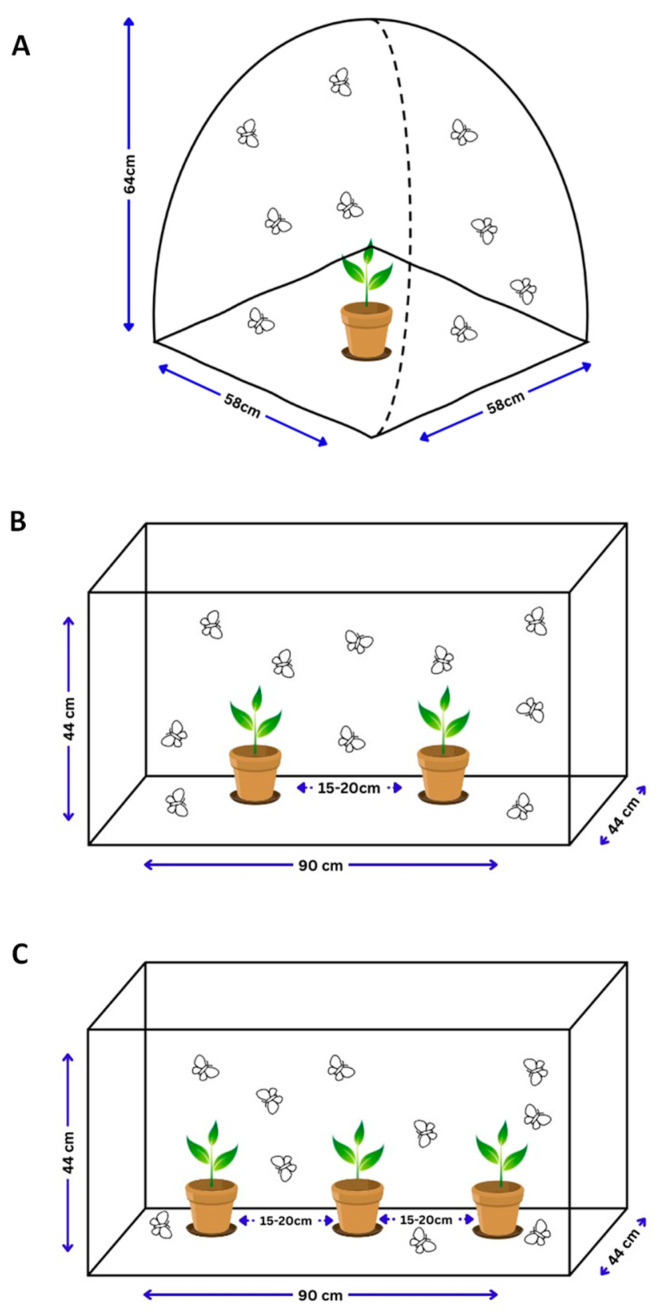
Schematic illustrations of the oviposition bioassays conducted under laboratory conditions. No-choice (**A**), two-choice (**B**), and multiple-choice (**C**) (note for multiple-choice: sunhemp (S), desmodium (D), maize (M), and cage walls (W); three plant arrangements were tested—S:D:M:W, D:M:S:W, and M:S:D:W).

The oviposition bioassays were performed under laboratory conditions and were modified according to the method described by Khan et al. [19] and Wang et al. [20] to investigate whether female moths display a preference for any particular host plant. The no-choice bioassays were performed using 58 W × 58 D × 64 H cm insect-rearing net tents (Figure 1A), whereas the two-choice and multiple-choice bioassays were performed in 90 L × 44 W × 44 H cm insect-rearing net cages (Figure 1B,C). For no-choice, a potted plant of each host plant with approximately equal leaf biomass was positioned in the center of each cage along with a feeding container with a 10% honey-sugar solution. In addition, an attempt was made to use plants of the same height and leaf biomass; however, this was complicated in some cases and can, therefore, be considered one of the limitations of this experiment. Next, five pairs of male and female moths (2–3 days old) were introduced into each cage and allowed to mate for 48 h. In the case of the two-choice bioassays, still following the procedures of the no-choice bioassays, potted plants of each host plant were placed at the center of each cage, approximately 15 cm apart from each other. Furthermore, our research aimed to investigate the effects of two repellent host plants, sunhemp (S) and desmodium (D), on maize (M) and cage walls (W) by conducting multiple-choice bioassays. We opted to assess sunhemp and desmodium because previous studies have documented their repelling properties. Additionally, our decision to examine sunhemp was driven by its notable attributes under field conditions, including high germination rate, rapid growth, and low maintenance requirements. In the case of desmodium, our decision was influenced by its extensive usage in various experiments across Africa and simply to reconfirm its reduced oviposition effects. Additionally, we placed particular emphasis on the arrangement of the individual host plants in the multiple-choice bioassays to mimic an intercropping scenario under laboratory conditions. This approach allowed us to observe the effects of these two host plants when intercropped with maize without having to physically grow plants in the field. Thus, three combinations were tested (S:D:M:W, D:M:S:W, and M:S:D:W). Each of the selected host plants was placed in the center of the cage equidistant from each other. Egg masses were collected from the plants and cage walls every 24 h (five consecutive days) with a fine brush, and the number of egg masses and eggs on both the plants and cage walls was counted. The number of eggs was counted using the ImageJ software (ImageJ 1.54 d; Wayne Rasband, National Institute of Health, Bethesda, MD, USA). Each egg mass was carefully separated into individual eggs with the use of two fine brushes. Subsequently, the separated eggs were placed in a petri dish, evenly distributed, and photographed. The captured image was then uploaded to ImageJ, where the “analyze particle” function was used to count the number of eggs. In each bioassay, the cage wall was considered as an ovipositing surface and was used as the control. Previous studies have shown that when *S. frugiperda* moths encounter unfavorable host plants, they tend to oviposit on alternative surfaces, such as cage walls, rather than on the plants themselves [20,21,22,23,24,25]. We used this behavioral pattern to assess plant acceptance of oviposition. To ensure reliability, the no-choice and two-choice bioassays were repeated three times, whereas the multiple-choice bioassays were replicated five times.

### 2.4. Data Analysis

The graphs and statistical analyses were performed using the GraphPad Prism software version 9.0 for Windows (GraphPad Software, San Diego, CA, USA). No-choice data were analyzed using an unpaired two-sample *t*-test, followed by multiple-choice tests (Fisher’s LSD). The data to construct the no-choice and two-choice percentage proportion graphs of egg masses and eggs were arcsine square root transformed for normalization and then analyzed using an unpaired two-sample *t*-test. On the other hand, the two-choice and multiple-choice data were analyzed using the analysis of variance (ANOVA), and mean separation was performed by Tukey’s at (*p* < 0.05). The means of the untransformed data were used to create the figures.

## 3. Results

The aim of the no-choice bioassays was to examine the oviposition behavior of adult *S. frugiperdas* under conditions in which they were restricted to either a single plant species or the cage wall (control). The two-choice bioassays were performed to gain further insight into the oviposition behavior of adult *S. frugiperdas* when presented with two different plants. Finally, the multiple-choice bioassays were designed to investigate three aspects: first, the simultaneous evaluation of three plants to assess their attractiveness to *S. frugiperda* moths; second, the influence of manipulating the position of each host plant on oviposition behavior; and finally, to mimic the effects of intercropping maize with sunhemp and desmodium.

### 3.1. No-Choice Bioassays (Number of Egg Masses)

**Figure 2:**
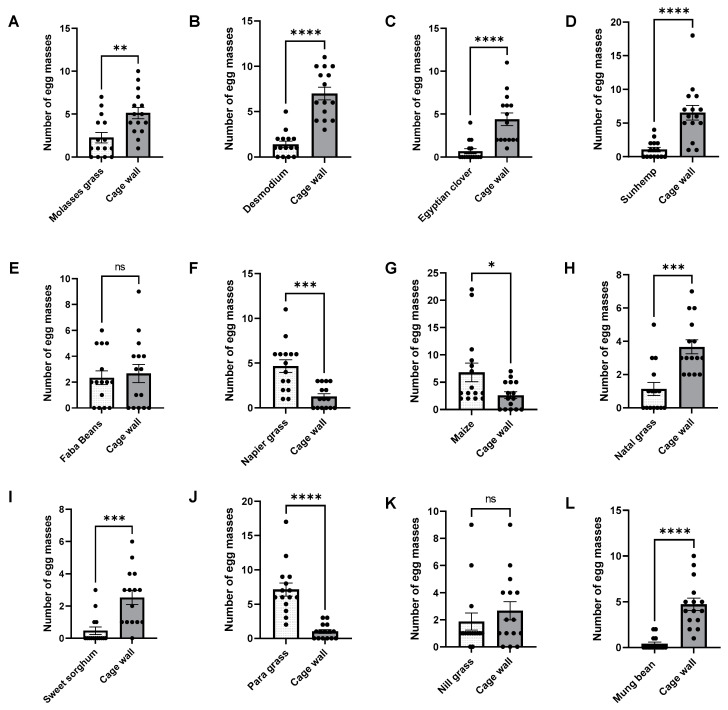
Number of egg masses in no-choice bioassays, under laboratory conditions, for molasses grass (**A**), desmodium (**B**), Egyptian clover (**C**), sunhemp (**D**), faba beans (**E**), napier grass (**F**), maize (**G**), natal grass (**H**), sweet sorghum (**I**), para grass (**J**), nill grass (**K**), and mung bean (**L**). Treatments that are significantly different by unpaired *t*-test are indicated by; ns, *p* > 0.05; *: *p* < 0.05; **: *p* < 0.01; ***: *p* < 0.001; and ****: *p* < 0.0001.

**Figure 3:**
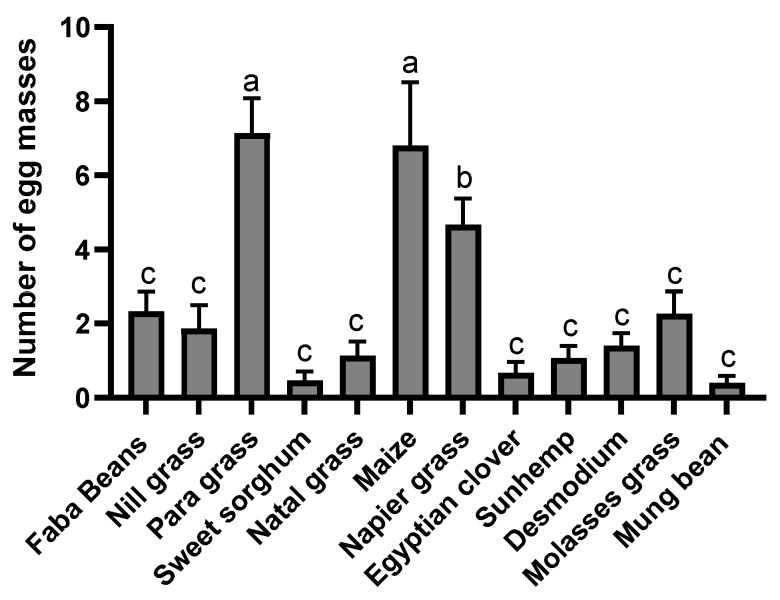
*Spodoptera frugiperda* egg masses oviposited in the no-choice bioassays. Twelve different host plants (faba beans, para grass, molasses grass, maize, desmodium, Egyptian clover, sweet sorghum, sunhemp, nill grass, natal grass, napier grass, and mung bean) were tested in no-choice comparisons for oviposition by mated adult *S. frugiperda* moths. Data are presented as mean ± SE. Means with the same letter are not significantly different according to Fisher’s LSD, *p* < 0.05.

In the no-choice bioassays, the highest number of egg masses was observed in para grass, followed by maize and napier grass, whereas mung bean, sweet sorghum, Egyptian clover, sunhemp, natal grass, desmodium, and molasses grass showed the lowest (Figure 2). Significantly higher numbers of egg masses were observed in maize (*t* = 2.3, *p* = 0.0291), napier grass (*t* = 4.317, *p* = 0.0002), and para grass (*t* = 6.167, *p* = 0.0001) than in the cage walls. In contrast, the number of egg masses in mung bean (*t* = 6.202, *p* = 0.0001), sunhemp (*t* = 4.833, *p* = 0.0001), desmodium (*t* = 7.184, *p* = 0.0001), molasses grass (*t* = 3.199, *p* = 0.0034), Egyptian clover (*t* = 4.623, *p* = 0.0001), sweet sorghum (*t* = 4.096, *p* = 0.0003), and natal grass (*t* = 4.417, *p* = 0.0001) was significantly lower than that in the cage wall. However, the average number of egg masses in the no-choice bioassays for faba beans (*t* = 0.3787, *p* = 0.7077) and nill grass (*t* = 0.8663, *p* = 0.3937) was not significantly different from that in the cage wall. Further analysis using ANOVA (multiple-choice tests and Fisher’s LSD test) (Figure 3) supports the above results.

There was a preference for these three host plants, particularly para grass, napier grass, and maize. In addition, there were reduced oviposition effects exhibited by sweet sorghum, natal grass, Egyptian clover, sunhemp, desmodium, molasses grass, and mung bean. However, strong reduced oviposition effects were exhibited by sweet sorghum, sunhemp, Egyptian clover, mung bean, desmodium, and natal grass.

### 3.2. No-Choice Bioassays (Number of Eggs)

**Figure 4:**
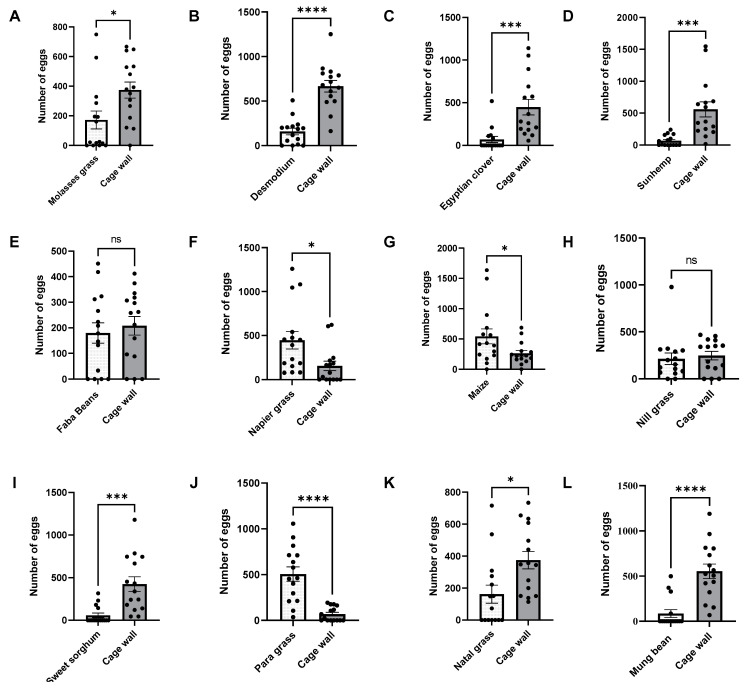
Number of eggs in no-choice bioassays, under laboratory conditions, for molasses grass (**A**), desmodium (**B**), Egyptian clover (**C**), sunhemp (**D**), faba beans (**E**), napier grass (**F**), maize (**G**), natal grass (**H**), sweet sorghum (**I**), para grass (**J**), nill grass (**K**), and mung bean (**L**). Treatments that are significantly different by unpaired *t*-test are indicated by; ns, *p* > 0.05; *: *p* < 0.05; ***: *p* < 0.001; and ****: *p* < 0.0001.

**Figure 5:**
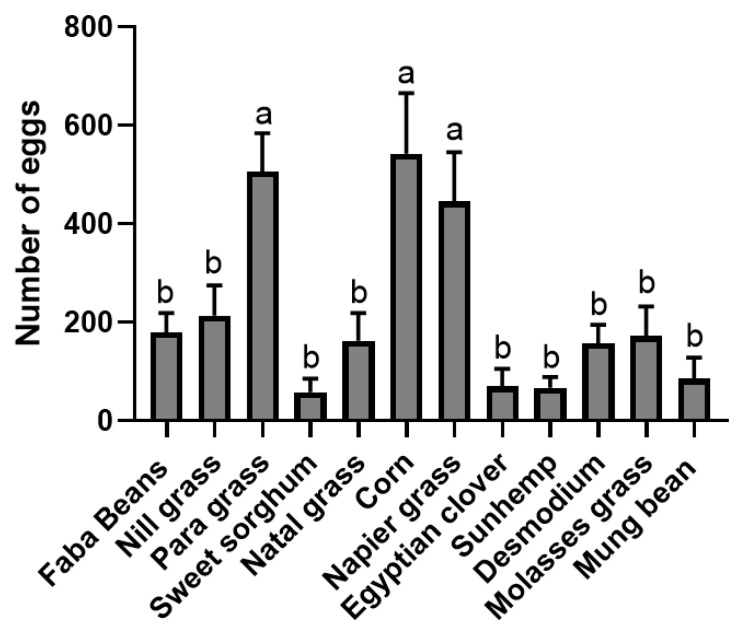
*Spodoptera frugiperda* eggs oviposited in the no-choice bioassays. Twelve different host plants (faba beans, para grass, molasses grass, maize, desmodium, Egyptian clover, sweet sorghum, sunhemp, nill grass, natal grass, napier grass, and mung bean) were tested in no-choice comparisons for oviposition by mated adult *S. frugiperda* moths. Data are presented as mean ± SE. Means with the same letter are not significantly different according to Fisher’s LSD, *p* < 0.05.

A similar trend was observed in the number of eggs. The highest number of eggs was observed on maize, para grass, and napier grass, while the lowest number of eggs was found in mung bean, sweet sorghum, sunhemp, Egyptian clover, desmodium, natal grass, and molasses grass (Figure 4). Significant differences were observed for napier grass (*t* = 2.569, *p* = 0.0158), maize (*t* = 2.109, *p* = 0.0440), and para grass (*t* = 5.430, *p* = 0.0001) than that in the cage wall. In contrast, the number of eggs for natal grass (*t* = 2.702, *p* = 0.0116), sweet sorghum (*t* = 4.055, *p* = 0.0004), sunhemp (*t* = 4.068, *p* = 0.0004), Egyptian clover (*t* = 3.852, *p* = 0.0006), mung bean (*t* = 5.105, *p* = 0.0001), and molasses grass (*t* = 2.494, *p* = 0.0188) were significantly lower than those of the cage wall, with the exception of nill grass (*t* = 0.4345, *p* = 0.6673) and faba beans (*t* = 0.5272, *p* = 0.6022). Further analysis using ANOVA (multiple-choice tests and Fisher’s LSD test) (Figure 5) also supported the above results. These results suggest that para grass and napier grass have potentially attractive properties, comparable to those of maize. Strong reduced oviposition effects were exhibited by sweet sorghum, sunhemp, Egyptian clover, mung bean, desmodium, and natal grass.

### 3.3. Attraction Ratios in No-Choice Bioassays (Number of Egg Masses and Number of Eggs)

**Figure 6:**
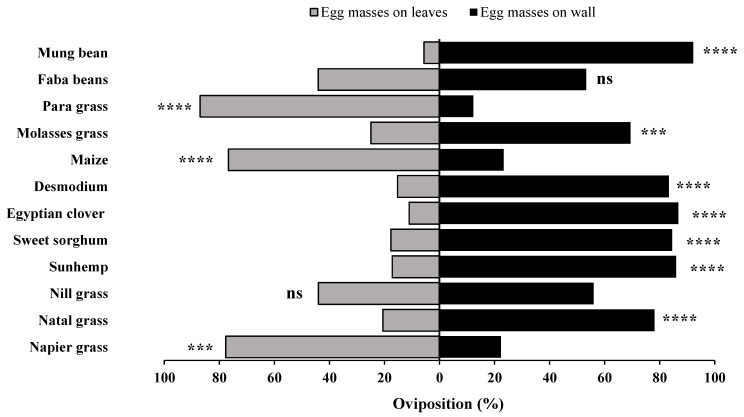
*Spodoptera frugiperda* percentage egg masses oviposited in no-choice bioassays. Twelve different host plants (faba beans, para grass, molasses grass, maize desmodium, Egyptian clover, sweet sorghum, sunhemp, nill grass, natal grass, napier grass, and mung bean) were tested in no-choice comparisons for oviposition by mated adult *S. frugiperda* moths. Treatments that are significantly different by unpaired *t*-test are indicated by; ns, *p* > 0.05; ***: *p* < 0.001; and ****: *p* < 0.0001.

**Figure 7:**
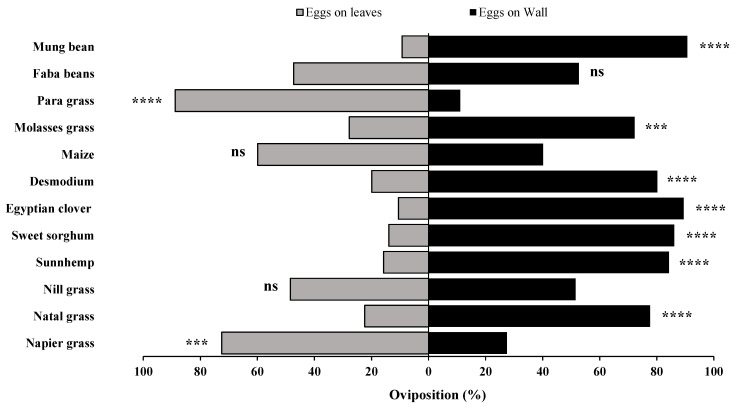
*Spodoptera frugiperda* percentage eggs oviposited in no-choice bioassays. Twelve different host plants (faba beans, para grass, molasses grass, maize desmodium, Egyptian clover, sweet sorghum, sunhemp, nill grass, natal grass, napier grass, and mung bean) were tested in no-choice comparisons for oviposition by mated adult *S. frugiperda* moths. Treatments that are significantly different by unpaired *t*-test are indicated by; ns, *p* > 0.05; ***: *p* < 0.001; and ****: *p* < 0.0001.

To further assess the effect of each host plant on the oviposition behavior of the *S. frugiperda* adults, the no-choice data for each host plant were converted into percentage attraction ratios (Figure 6 and Figure 7). The highest proportions of egg masses and eggs were oviposited on para grass (86.9%, 88.8%), napier grass (77.7%, 72.5%), and maize (76.7%, 59.9%), followed by faba beans (44.1%, 47.3%), nill grass (44.0%, 48.5%), molasses grass (24.9%, 27.8%), natal grass (20.5%, 22.4%), desmodium (15.3%, 19.8%), sweet sorghum (17.7%, 13.9%), sunhemp (17.2%, 15.7%), and Egyptian clover (11.1%, 10.6%). Furthermore, significant differences were observed among the tested host plants, specifically for para grass (*t* = 6.167, *p* = 0.0001), napier grass (*t* = 4.317, *p* = 0.0002), maize (*t* = 2.3, *p* = 0.0291), molasses grass (*t* = 3.199, *p* = 0.0034), natal grass (*t* = 4.417, *p* = 0.0001), desmodium (*t* = 7.184, *p* = 0.0001), sweet sorghum (*t* = 4.096, *p* = 0.0003), sunhemp (*t* = 4.833, *p* = 0.0001), Egyptian clover (*t* = 4.623, *p* = 0.0001), and mung bean (*t* = 4.623, *p* = 0.0001), except for faba beans (*t* = 0.3787, *p*= 0.7077) and nill grass (*t* = 0.8663, *p* = 0.3937). The results indicate that in terms of percentage egg masses and the number of eggs, para grass, maize, and napier grass were highly preferred by the *S. frugiperda* adults, whereas Egyptian clover, sunhemp, sweet sorghum, desmodium, natal grass, and molasses grass were the least preferred by the *S. frugiperda* adults.

### 3.4. Two-Choice Bioassays (Number of Egg Masses and Number of Eggs)

**Figure 8:**
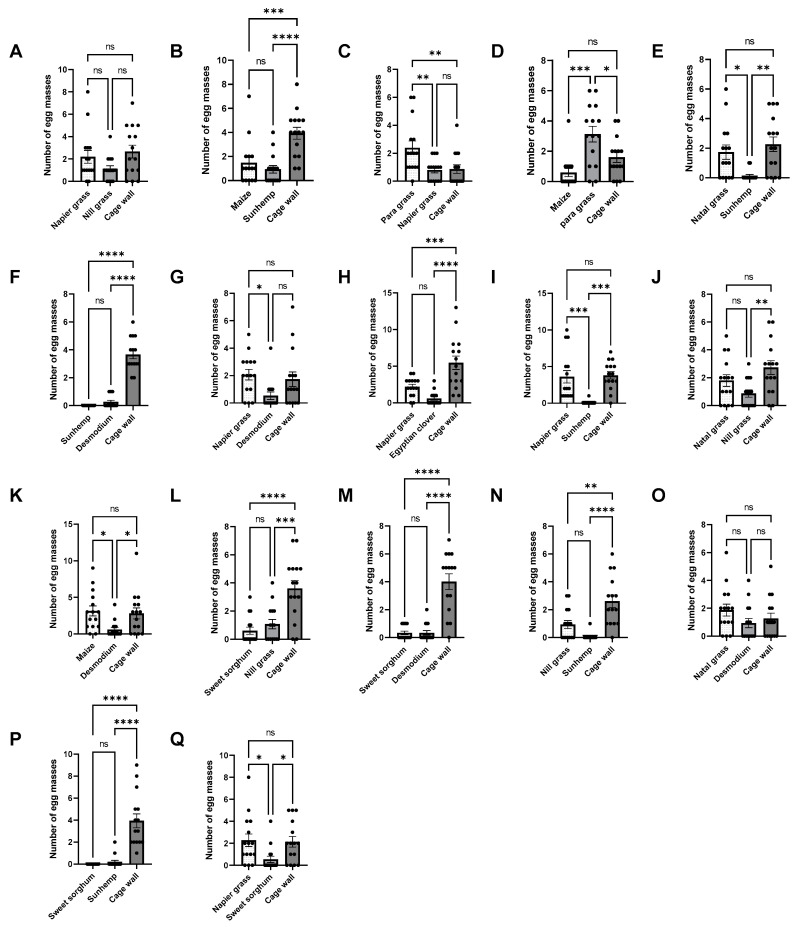
Number of egg masses in two-choice bioassays, under laboratory conditions, for napier grass and nill grass (**A**), maize and sunhemp (**B**), para grass and napier grass (**C**), maize and para grass (**D**), natal grass and sunhemp (**E**), sunhemp and desmodium (**F**), napier grass and desmodium (**G**), napier grass and Egyptian clover (**H**), napier napier grass and sunhemp (**I**), natal grass and nill grass (**J**), maize and desmodium (**K**), sweet sorghum and nill grass (**L**), sweet sorghum and desmodium (**M**), nill grass and sunhemp (**N**), natal grass and desmodium (**O**), sweet sorghum and sunhemp (**P**), napier napier grass and sweet sorghum (**Q**). Treatments that are significantly different by Tukey’s post hoc test are indicated by; ns, *p* > 0.05; *: *p* < 0.05; **: *p* < 0.01; ***: *p* < 0.001; and ****: *p* < 0.0001.

**Figure 9:**
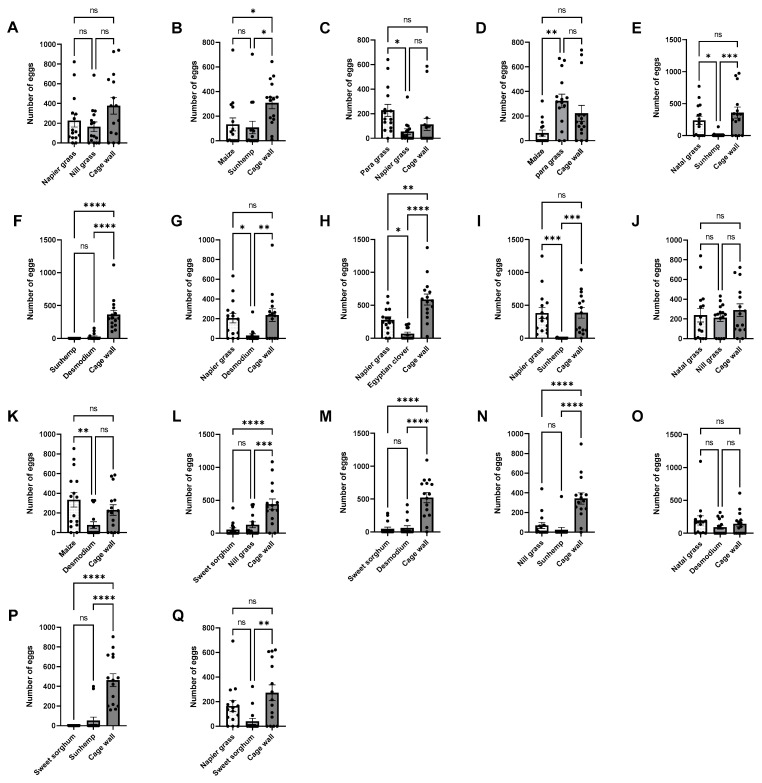
Number of eggs in two-choice bioassays, under laboratory conditions, for napier grass and nill grass (**A**), maize and sunhemp (**B**), para grass and napier grass (**C**), maize and para grass (**D**), natal grass and sunhemp (**E**), sunhemp and desmodium (**F**), napier grass and desmodium (**G**), napier grass and Egyptian clover (**H**), napier grass and sunhemp (**I**), natal grass and nill grass (**J**), maize and desmodium (**K**), sweet sorghum and nill grass (**L**), sweet sorghum and desmodium (**M**), nill grass and sunhemp (**N**), natal grass and desmodium (**O**), sweet sorghum and sunhemp (**P**), and napier grass and sweet sorghum (**Q**). Treatments that are significantly different by Tukey’s post hoc test are indicated by; ns, *p* > 0.05; *: *p* < 0.05; **: *p* < 0.01; ***: *p* < 0.001; and ****: *p* < 0.0001.

Host plant preference was further examined for various other combinations (Figure 8 and Figure 9). The recorded data on the number of egg masses and eggs for each plant combination, along with the control represented by the cage wall, provided valuable insights into the oviposition preferences of *S. frugiperda.*

Napier grass, para grass, natal grass, maize, and cage wall consistently attracted more egg masses and eggs than several other plants, including nill grass, desmodium, Egyptian clover, sunhemp, and sweet sorghum. Moreover, significant differences in egg masses were observed between the following combinations; para grass and grass (*F* = 6.64, *p* = 0.0031), maize and para grass (*F* = 10.80, *p* = 0.0002), natal grass and sunhemp (*F* = 7.64, *p* = 0.0015), napier grass and desmodium (*F* = 3.80, *p* = 0.0302), napier grass and sunhemp (*F* = 13.6, *p* = 0.0001), maize and desmodium (*F* = 5.12, *p* = 0.0103), and napier grass and sweet sorghum (*F* = 4.30, *p* = 0.0200) (Figure 8). Additionally, significant differences were observed in the number of eggs between the following combinations: para grass and napier grass (*F* = 4.36, *p* = 0.0191), maize and para grass (*F* = 6.60, *p* = 0.0032), natal grass and sunhemp (*F* = 8.006, *p* = 0.0011), napier grass and desmodium (*F* = 5.60, *p* = 0.0070), napier grass and Egyptian clover (*F* = 20.9, *p* = 0.0001), napier grass and sunhemp (*F* = 10.6, *p* = 0.0002), and maize and desmodium (*F* = 5.26, *p* = 0.0092).

These results suggested that different host plants have varying levels of attractiveness and repellency across combinations. Furthermore, when any of the plants with high reduced oviposition effects, such as sunhemp, Egyptian clover, and sweet sorghum, were tested against the attractant plants (maize and napier grass), it was observed that the *S. frugiperda* moths laid more eggs on the cage wall. Notably, when desmodium was tested against maize, napier grass, and natal grass, a consistent pattern was observed when more egg masses were deposited on the attractant plant, even though the attractant plant was adjacent to the repellent plant. For sunhemp, when tested against maize, natal grass, napier grass, and nill grass, more egg masses were deposited on the cage wall. Moreover, the control (cage wall) served as a reference point, indicating that in general, the insects preferred the conditions provided by the cage wall over specific plants, such as sunhemp, desmodium, Egyptian clover, and sweet sorghum.

### 3.5. Attraction Ratios in Two-Choice Bioassays (Number of Egg Masses and Number of Eggs)

**Figure 10:**
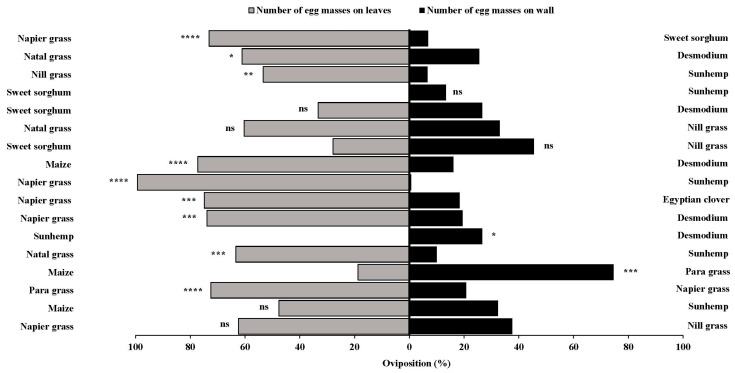
*Spodoptera frugiperda* percentage egg masses oviposited in two-choice bioassays. Twelve different combinations were tested in two-choice comparisons for oviposition by mated adult *S. frugiperda* moths. Combinations that are significantly different by unpaired *t*-test are indicated by; ns, *p* > 0.05; *: *p* < 0.05; **: *p* < 0.01; ***: *p* < 0.001; and ****: *p* < 0.0001.

**Figure 11:**
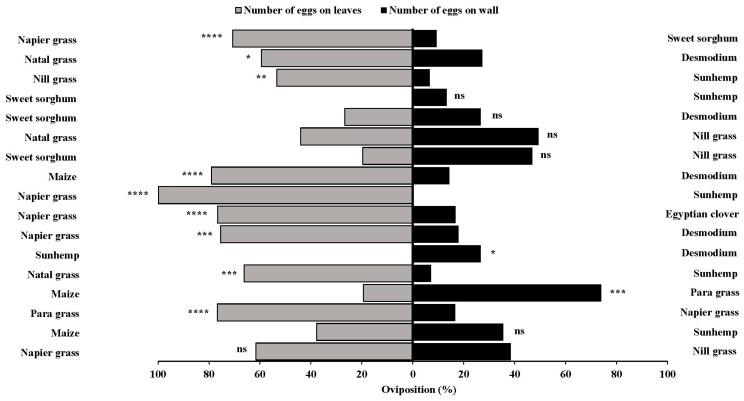
*Spodoptera frugiperda* percentage eggs oviposited in two-choice bioassays. Twelve different combinations were tested in two-choice comparisons for oviposition by mated adult *S. frugiperda* moths. Treatments that are significantly different by unpaired *t*-test are indicated by; ns, *p* > 0.05; *: *p* < 0.05; **: *p* < 0.01; ***: *p* < 0.001; and ****: *p* < 0.0001.

The two-choice bioassays revealed a consistent oviposition preference by the *S. frugiperda* moths across various plant combinations (Figure 10 and Figure 11). Napier grass consistently emerged as a highly preferred host for *S. frugiperda* than several other host plants, such as sunhemp, Egyptian clover, desmodium, sweet sorghum, and nill grass, attracting both egg masses and eggs. Similarly, maize exhibited strong attractiveness over desmodium, but only marginally when compared to sunhemp, in terms of both egg masses and eggs. Para grass also demonstrated higher attractiveness than napier grass and maize in its ability to attract egg masses and eggs. In contrast, desmodium, Egyptian clover, sweet sorghum, and sunhemp were consistently the least preferred among several other host plants.

Significant differences were observed in egg masses, particularly for para grass and napier grass (*t* = 4.90, *p* = 0.0001), napier grass and sweet sorghum (*t* = 5.63, *p* = 0.0001), nill grass and sunhemp (*t* = 3.13, *p* = 0.0041), maize and desmodium (*t* = 4.87, *p* = 0.0001), napier grass and sunhemp (*t* = 50.4, *p* = 0.0001), napier grass and Egyptian clover (*t* = 4.46, *p* = 0.0001), napier grass and desmodium (*t* = 3.72, *p* = 0.0009), sunhemp and desmodium (*t* = 2.256, *p* = 0.0320), natal grass and sunhemp (*t* = 3.71, *p* = 0.0009), and maize and para grass (*t* = 4.35, *p*= 0.0002). Differences were also observed in the number of eggs, specifically for napier grass and sweet sorghum (*t* = 5.02, *p* = 0.0001), maize and desmodium (*t* = 5.19, *p* = 0.0001), napier grass and sunhemp (*t* = 98.8, *p*= 0.0001), napier grass and Egyptian clover (*t* = 4.74, *p* = 0.0001), napier grass and desmodium (*t* = 3.95, *p* = 0.0005), natal grass and sunhemp (*t* = 4.09, *p* = 0.0003), maize and para grass (*t* = 4.27, *p* = 0.0002), and para grass and napier grass (*t* = 5.85, *p* = 0.0001). The results of this study demonstrated that vegetation selection has a significant influence on the oviposition preference of *S. frugiperda* moths. Furthermore, it is evident that the *S. frugiperda* moths showed a strong attraction, particularly to para grass, maize, and napier grass, whereas most other host plants, such as desmodium, sweet sorghum, sunhemp, natal grass, and Egyptian clover, were less attractive to the *S. frugiperda* moths.

### 3.6. Multiple-Choice Bioassays (Number of Egg Masses and Number of Eggs)

**Figure 12:**
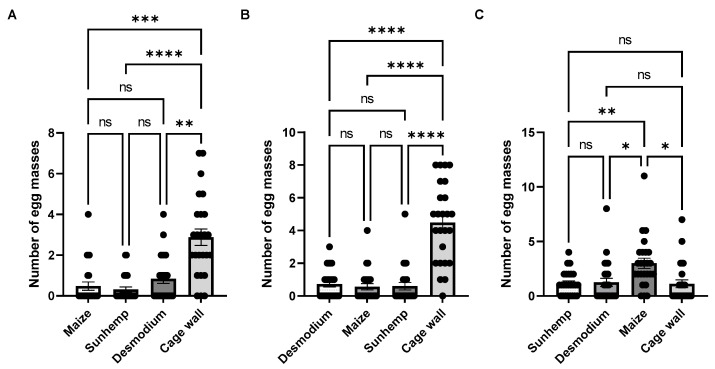
Number of egg masses in multiple-choice bioassays, under laboratory conditions, for maize, sunhemp, desmodium, and cage wall (M:S:D:W) (**A**); desmodium, maize, sunhemp, and cage wall (D:M:S:W) (**B**); and sunhemp, desmodium, maize, and cage wall (S:D:M:W) (**C**). Data are presented as mean ± SE. Treatments that are significantly different by Tukey’s post hoc test are indicated by; ns, *p* > 0.05; *: *p* < 0.05; **: *p* < 0.01; ***: *p* < 0.001; and ****: *p* < 0.0001.

**Figure 13:**
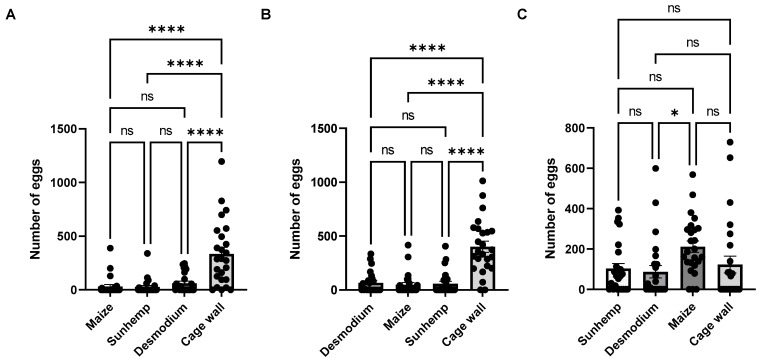
Number of eggs in multiple-choice bioassays, under laboratory conditions, for maize, sunhemp, desmodium, and cage wall (M:S:D:W) (**A**); desmodium, maize, sunhemp, and cage wall (D:M:S:W) (**B**); and sunhemp, desmodium, maize, and cage wall (S:D:M:W) (**C**). Data are presented as mean ± SE. Combinations that are significantly different by Tukey’s post hoc test are indicated by; ns, *p* > 0.05; *: *p* < 0.05 and ****: *p* < 0.0001.

To further assess the effects of the two repellent plants, a multiple-choice test was designed to compare the effects of desmodium and sunhemp against maize and cage walls (Figure 12 and Figure 13). The positioning effect of the repellent plants and maize was evaluated by randomly interchanging the positions of each combination. This randomized design allowed for the comprehensive observation of the individual effects of each repellent host plant on maize.

The number of egg masses for M:S:D:W (maize, sunhemp, desmodium, and cage wall) was the highest for W, followed by D, M, and S. For D:M:S:W (desmodium, maize, sunhemp, and cage wall), the number of egg masses was the highest for W, followed by D, M, and S. For S:D:M:W (sunhemp, desmodium, maize, and cage wall), the number of egg masses was the highest for M, followed by D, S, and W.

For M:S:D:W and D:M:S:W, significant differences in the number of egg masses were observed only when M, S, and D were compared against W. For S:D:M:W, significant differences were observed when S and D were compared to M and when M was compared to W (M:S:D:W: *F* = 19.8, *p* < 0.0001; D:M:S:W: *F* = 42.3, *p* < 0.0001; S:D:M:W: *F* = 5.83, *p* < 0.0027). For M:S:D:W and D:M:S:W, significant differences in the number of eggs were observed when M, S, and D were compared to W. For S:D:M:W, a significant difference was observed when D was compared to M (M:S:D:W: *F* = 19.7, *p* < 0.0001; D:M:S:W: *F* = 30.6, *p* < 0.0001; S:D:M:W: *F* = 3.06, *p* < 0.0320).

In all the instances, when sunhemp was placed next to maize, this consistently resulted in increased egg-laying by *S. frugiperda* on the cage walls, except when desmodium was placed next to maize. Interestingly, when desmodium was tested against an attractant plant (napier grass and maize), maize had higher egg masses and eggs, similar to the results of the two-choice tests. These results suggest that sunhemp may have a better reduced oviposition effect than desmodium when placed next to maize.

## 4. Discussion

In this study, the host plants found in Taiwan with potential deterrent or stimulating oviposition effects against *S. frugiperda* adults were selected and tested using oviposition preference bioassays.

The no-choice bioassays (oviposition preference bioassays and the percentage proportion of egg masses and eggs) revealed that the *S. frugiperda* moths oviposited egg masses and eggs on all the host plants tested in this experiment. In addition, napier grass, para grass, and maize stimulated oviposition, as evidenced by an increase in egg mass and the number of eggs laid. The attractiveness of napier grass and para grass is attributed to the release of the volatile compounds found in maize, and compounds such as hexane, hexenal, 3–hexen–1–ol, and acetate have been observed in napier grass or sorghum to produce a higher attraction to stem borers [26,27,28]. While our results demonstrate strong oviposition preferences for certain grass species, particularly napier grass and para grass, it’s important to note that oviposition preference doesn’t necessarily correlate with larval performance. Chen et al. [29] found that host plant suitability for larval development can vary significantly among these grass species, suggesting that *S. frugiperda* females may sometimes choose oviposition sites that are not optimal for offspring survival. This disconnect between adult preference and larval performance merits further investigation in future studies.

This is consistent with previous findings by [19,30,31] when assessing napier grass in both no-choice and two-choice bioassays. In the case of para grass, a strong preference was observed during the no-choice and two-choice bioassays. Previous studies have conducted oviposition assays with different species of *Brachiaria* grass, and have shown similar results [30,32,33,34,35]. To our knowledge, the invasive species found in Taiwan have not been studied. Sunhemp, desmodium, molasses grass, Egyptian clover, sweet sorghum, natal grass, and mung bean all exhibited reduced oviposition effects, except for faba beans and nill grass, in terms of the number of egg masses and number of eggs (oviposition preference bioassays and the percentage proportion of egg masses and eggs). Interestingly, sunhemp exhibited a reduced oviposition efffect, which coincides with Meagher et al. [36] and Guera et al. [37], whereby *S. frugiperda* adults were less attracted to oviposit on sunhemp. According to Ali and Wright [38], the presence of triterpenes, alkaloids, flavonoids, and phenolic substances in the leaves of sunhemp makes it repellent for ovipositing *S. frugiperda* adults. In the case of desmodium, it is a deterrent to stemborer moths and possibly *S. frugiperda* females because it produces repellent volatiles, such as (E)–β–ocimene and (E)–4,8–dimethyl–1,3,7–nonatriene. These compounds are responsible for the plant’s ability to repel certain moth species [39]. Next, molasses grass and natal grass belong to the same genus. They showed a reduced oviposition effect, which can be attributed to the bioactive compounds found in molasses grass, such as (E)–b–ocimene, a–terpinolene, b–caryophyllene, humulene, and (E)–4,8–dimethyl–1,3,7–nonatriene (DMNT) [30,39]. In the case of Egyptian clover, we speculate that the reduced oviposition effects are due to monoterpenoids and fatty acids, specifically 1–octen–3–ol. Previous studies have reported the oviposition deterrent effects of 1–octen–3–ol [40,41]. We can argue that the above Green Leaf Volatiles (GLVs) could be responsible for the reduced oviposition effects that Egyptian clover showed during the no-choice and two-choice oviposition bioassays. Furthermore, mung beans are mainly composed of aldehydes and alcohols, with benzaldehyde being detected more often than the other compounds [42]. We speculate that these compounds are responsible for the reduced oviposition effects observed in the no-choice bioassays. Benzaldehyde has been reported to have a repellent effect on several species of moths [43], and has also been reported to have repellent properties against *Drosophila melanogaster* (Meigen) (Diptera: Drosophilidae) [44]. However, contrary to the above, other experiments have reported that benzaldehyde was attractive as a single chemical or as blends of various lepidopteran species [45,46,47]. Sweet sorghum is primarily composed of alkanes, aldehydes, ketones, alcohols, esters, acids, phenols, and terpenes [48]. It is possible that any or a combination of these compounds is responsible for the reduced oviposition effects observed during the no-choice and two-choice bioassays. These results are consistent with those of the previous studies that conducted oviposition assays on these plants [24,30,36,49], except for Egyptian clover, nill grass, and natal grass. However, no significant differences were observed for faba bean and nill grass. Our no-choice bioassay for faba beans differed from that of Liu et al. [22], who reported that ovipositing *S. frugiperda* females showed no preference for faba beans in a two-choice bioassay. To the best of our knowledge, this is the only study to date that has tested faba beans. Therefore, further testing would be helpful to substantiate these findings.

The results of the two-choice bioassays for the number of egg masses and number of eggs (oviposition preference bioassays and the percentage proportion of egg masses and eggs) varied across combinations. The two-choice bioassays further support the notion that sunhemp, sweet sorghum, Egyptian clover, natal grass, and desmodium have repellent properties, whereas napier grass, para grass, and maize have attractive properties for *S. frugiperda* females. The results for desmodium and maize are in line with the findings of Sobhy et al. [24] and Peter et al. [50]; however, the results also contradict those of Erdei et al. [51] when they compared maize against desmodium. No significant differences were observed in the number of egg masses or eggs. In addition, the present study found that when evaluating highly repellent plants, such as sunhemp, desmodium, Egyptian clover, and sweet sorghum, the *S. frugiperda* moths consistently deposited more egg masses and eggs on the cage wall (except for desmodium against napier grass) than the attractant plants, such as maize and napier grass. A possible explanation for this may be the strong reduced oviposition effects of the host plants. For instance, the fact that the *S. frugiperda* moths chose to lay more on the cage wall despite having a highly attractive host plant suggests that when any of the highly repellent plants were in proximity, they successfully repelled the *S. frugiperda* moths away from the attractant plant. Another interesting finding was that when desmodium was tested against attractant plants such as napier grass and maize, the *S. frugiperda* moths consistently laid more on the attractant plants, whereas when sunhemp was in proximity to the attractant plants, the *S. frugiperda* moths consistently laid more on the cage wall. A possible explanation might be that sunhemp has a stronger reduced oviposition effect than desmodium. Furthermore, our findings highlight the complex dynamics of neighboring plant interactions in *S. frugiperda* oviposition behavior. Notably, when the repellent plants were placed near the highly attractive plants, we observed varying degrees of reduction in egg deposition on the repellent plants themselves. This suggests that the presence of attractive plants may enhance the reduced oviposition effect of repellent plants by providing moths with a clear alternative oviposition site. However, this effect appeared to be plant-specific; for instance, while sunhemp maintained strong reduced oviposition effects even in proximity to the attractive plants, desmodium’ s reduced oviposition seemed to be partially masked when placed near maize. This differential response to neighboring plants could be attributed to variations in the strength and nature of the volatile signals emitted by different plant combinations, as well as potential synergistic or antagonistic interactions between plant volatiles in close proximity. Moreover, we speculate that the volatiles released by maize tend to mask the repellent volatiles of desmodium, but not of sunhemp. It has been reported that specific herbivorous insects exhibit altered host plant location abilities only when the mixing of host and non-host volatiles reaches a particular threshold. This threshold-dependent phenomenon led to observable masking effects. However, if mixing different odor plumes fails to surpass this threshold, masking effects are unlikely to occur [52,53]. In our study, masking effects likely occurred when sunhemp was in close proximity to the attractant plants, whereas the opposite was observed when desmodium was in close proximity to the attractant plants.

The results for sunhemp in the two-choice bioassays align with the findings of Meagher et al. [36] and Guera et al. [37], who conducted multiple-choice bioassays against maize. In regard to sweet sorghum, while it was not directly compared to maize, it exhibited lower oviposition of egg masses and eggs when compared to napier grass and desmodium. These findings are consistent with a previous study by Rebe et al. [49], who compared sweet sorghum to maize, with the exception of sweet sorghum and desmodium, for which no previous studies have been documented. Interestingly, from our results, para grass can be considered a novel attractant plant, whereas mung bean and Egyptian clover are novel repellent plants. This was further confirmed when para grass was compared to maize, the naturally preferred host plant of *S. frugiperda*, and when Egyptian clover was compared to napier grass. The genus *Brachiaria* is widespread in Taiwan and other regions worldwide. Several studies have examined *Brachiaria* species, especially *Brachiaria brizantha* (Hochst. ex A. Rich., R. Webster) (Poales: Poaceae) and *Brachiaria ruziziensis* (Germ. and Evrard) (Poales: Poacea) in “push-pull strategy” field trials and oviposition preference bioassays [54,55,56,57]. To our knowledge, para grass has not been studied or reported in oviposition preference bioassays nor “push-pull strategy” field trials. Hence, these findings suggest that para grass could potentially serve as a “pull” plant in future push-pull systems. Additionally, the volatile compounds emitted by para grass should be explored and considered for future oviposition bioassay trials. Furthermore, the results can be used to raise awareness about this grass among small farmers across Taiwan and other parts of the world. Recommendations, such as removing this grass from nearby potential maize growing areas during the off-season, may lead to a decrease in the *S. frugiperda* population, thus forcing its migration to alternative areas with suitable hosts for the continuation of its cycle.

In the final experiment, a combination of sunhemp, desmodium, and maize was tested to observe the effects of both repellent crops against *S. frugiperda*. The results indicated that when both repellent plants were used simultaneously, the *S. frugiperda* moths preferred to oviposit more egg masses and eggs on the cage wall surfaces, except for the S:D:M:W combination, where maize was placed next to desmodium; interestingly, the *S. frugiperda* moths chose to lay more on maize than sunhemp, desmodium, and the cage wall. This finding follows the same trend as the previous results of the no-choice and two-choice experiments, which revealed that desmodium does not necessarily repel *S. frugiperda* moths away from maize. It is possible that certain volatile compounds emitted by maize could mask the repellent odors produced by desmodium. In addition, a synergistic effect between maize and desmodium odors could explain the observed preference for maize. Another explanation could be the positioning of the plants in the experimental setting, which could have altered the oviposition preferences of the *S. frugiperda* moths. For example, if the maize plant was placed in a way that made it more accessible or appealing to the moths, they may have opted to lay more eggs on it than on the other plants. It is also likely that the concentration and potency of repellent volatiles differed between the plants, and the *S. frugiperda* moths were able to detect these minute differences. For example, it is possible that the repellent component concentration in desmodium was insufficient to deter the moths from laying eggs on the surrounding plants. Another possible explanation could be olfactory masking. Lastly, it could happen if there were any slight differences in the growth of the plants during the experimental period. If the maize plants were slightly more vigorous than the repellent plants, the *S. frugiperda* moths might have been more attracted to maize because of factors related to plant health.

Notably, across the no-choice, two-choice, and multiple-choice bioassays, the *S. frugiperda* females in all the combinations oviposited on every tested host plant and cage wall surface. These observations suggest that *S. frugiperda* females are not strongly attracted to either of the plant options, or that the mixture of plant volatiles inside the cage affects their decision making when exposed to a more complex host range [20,22,23,24,25,58]. Instead, they choose to lay their eggs on a neutral surface such as a cage wall, which may not provide suitable conditions for egg development. Da Silva et al. [59] and Jones and Sparks [27] previously reported this behavior, noting that large numbers of *S. frugiperda* can deposit their eggs on non-host plants and objects. This indiscriminate oviposition behavior of *S. frugiperda* was previously reported by Rojas et al. [60], who observed numerous moths laying their eggs on irregular surfaces, suggesting that proximity might play a significant role in determining the choice of oviposition location. The presented findings contradict the established “mother knows best principle” or “optimal oviposition theory,” which has been extensively discussed in previous research [61,62,63,64]. This theory is based on the notion that offspring have limited ability to select their developmental environment during their juvenile stages. Hence, it is believed that it becomes the mother’s responsibility to locate a suitable host that ensures the survival and development of the offspring [65]. If we place this in context, the goal of this theory is to ensure the survival of the offspring, and a better and more suitable environment would guarantee this. However, there are still a myriad of factors that require further research to explain such behavior in *S. frugiperda* females.

Oviposition preference assays, specifically for *S. frugiperda,* have not been reported previously in Taiwan. Hence, our research serves as a reference point for future research that might engage in this field. These results should be considered when selecting attractant or repellent crops for the establishment of push-pull strategy in Taiwan, Asia, and possibly other parts of the world.

It is beyond the scope of this study to test a larger number of potential host plants and replicate each no-choice and two-choice bioassay more than three times. Certainly, a higher number of replicates for each host plant would have complemented the data obtained from this experiment; nevertheless, the data collected were modified to be collected every 24 h for five days, thus compensating for the number of replicates. Moreover, in some cases, when the adults were initially mated, some died during the five-day observation period, which delayed some of the results. Also, another important limitation of the current study is that we did not directly observe the behavioral mechanisms underlying the observed oviposition patterns. While we have characterized the distribution of *S. frugiperda* eggs across different individual and plant combinations, additional behavioral experiments would be needed to definitively determine whether female moths are actively avoiding plants with lower egg numbers, or simply exhibiting preferential oviposition on more attractive hosts.

Future studies could focus on multiple-choice bioassays, volatile compounds, and olfactometry experiments to further explore the oviposition behavior of *S. frugiperda* adults under laboratory conditions. However, in the meantime, napier grass, para grass, desmodium, molasses grass, natal grass, Egyptian clover, sweet sorghum, sunhemp, and mung bean have the potential for further studies, either under field or laboratory conditions.

## 5. Conclusions

The identification and validation of repellent and attractant host plants are crucial for developing effective integrated pest management strategies, particularly push-pull strategies that minimize pesticide use while ensuring sustainable maize production. This study provides the first comprehensive assessment of *S. frugiperda* oviposition preferences among leguminous and poaceous plants in Taiwan, contributing to the potential implementation of push-pull strategies. Our findings revealed clear hierarchies in host plant preference: para grass, maize, and napier grass emerged as highly attractive hosts, while several other tested plants demonstrated significant reduced oviposition effects. The two-choice and multiple-choice bioassays further established varying degrees of attraction and repellency among different plant combinations, suggesting potential for strategic intercropping applications. These findings align with global sustainability initiatives, particularly supporting the UN’s 2030 Sustainable Development Goals of zero hunger, climate action, and responsible consumption and production. While our study focused on a select group of locally available host plants, it establishes a foundation for future research in Taiwan and potentially in other countries. Further investigation of additional host plants, particularly those exhibiting strong reduced oviposition effects, could enhance our understanding of *S. frugiperda* behavior and contribute to the development of more effective, environmentally sustainable pest management strategies.

## Data Availability

The datasets used and/or analyzed during the current study are available from the corresponding author upon reasonable request.
